# Intravenous ascorbic acid as an adjuvant to interleukin-2 immunotherapy

**DOI:** 10.1186/1479-5876-12-127

**Published:** 2014-05-13

**Authors:** Samuel C Wagner, Boris Markosian, Naseem Ajili, Brandon R Dolan, Andy J Kim, Doru T Alexandrescu, Constantin A Dasanu, Boris Minev, James Koropatnick, Francesco M Marincola, Neil H Riordan

**Affiliations:** 1 Batu Biologics, San Diego, California, USA; 2 Moores UCSD Cancer Center, University of California San Diego, San Diego, USA; 3 Genelux Corporation, San Diego Science Center, San Diego, California, USA; 4 Division of Neurosurgery, University of California San Diego, San Diego, USA; 5 Department of Hematology and Oncology, University of Connecticut, Hartford, Connecticut, USA; 6 Lawson Health Research Institute and Department of Oncology, The University of Western Ontario, London, Ontario, Canada; 7 Sidra Medical and Research Center, Doha, Qatar; 8 Riordan Clinic, Wichita, Kansas, USA; 9 Vaxenta Inc, San Diego, California, USA

**Keywords:** Interleukin-2, Cancer immunotherapy, Intravenous ascorbic acid, T cell, Natural killer

## Abstract

Interleukin-2 (IL-2) therapy has been demonstrated to induce responses in 10-20% of advanced melanoma and renal cell carcinoma patients, which translates into durable remissions in up to half of the responsers. Unfortunately the use of IL-2 has been associated with severe toxicity and death. It has been previously observed and reported that IL-2 therapy causes a major drop in circulating levels of ascorbic acid (AA). The IL-2 induced toxicity shares many features with sepsis such as capillary leakage, systemic complement activation, and a relatively non-specific rise in inflammatory mediators such as TNF-alpha, C-reactive protein, and in advanced cases organ failure. Animal models and clinical studies have shown rapid depletion of AA in conditions of sepsis and amelioration associated with administration of AA (JTM 9:1-7, 2011). In contrast to other approaches to dealing with IL-2 toxicity, which may also interfere with therapeutic effects, AA possesses the added advantage of having direct antitumor activity through cytotoxic mechanisms and suppression of angiogenesis. Here we present a scientific rationale to support the assessment of intravenous AA as an adjuvant to decrease IL-2 mediated toxicity and possibly increase treatment efficacy.

## Background

The basis of cancer therapy is to identify/develop interventions that: a) selectively kill tumor cells while sparing non-malignant cells; b) prevent development of tumor-resistance; and c) act systemically to prevent relapse. Theoretically, immunotherapy of cancer would achieve all these aims. Selective killing of tumor cells has been demonstrated by a wide range of immune cells ranging from conventional CD8 T cells, gamma delta T cells, natural killer (NK) cells, natural killer T (NKT) cells and in some studies neutrophils. Other components of the immune system expressing tumor selectivity include complement factors and natural antibodies of the IgM isotype. Tumor resistance to immunotherapy, differs from resistance to chemotherapy, where expression of multiple drug resistance proteins actively pumps out cancer-toxic substances. One mechanism is downregulation of human leukocyte antigen (HLA) by tumor cells in response to T cell killing. The immune system conceptually overcomes this by the NK subset which preferentially kills cells with downregulated HLA. The other mechanism of tumor resistance from immunological attack is by mutating antigens that are being recognized. Given the promiscuous binding ability of T cell and B cell receptors to bind antigens, the cells could conceptually “mutate with the tumor” in order to recognize and kill cells with variant antigens. Immunological destruction of neoplasia is believed to occur at a systemic level and thus arises the possibility to inhibit metastasis and tumor recurrence, in part through induction of immunological memory.

The history of tumor immunotherapy begins with the work of William Coley who induced a systemic inflammatory/immune activation through administration of killed S. pyogenes and Serratia marcescens bacteria in patients with soft tissue sarcoma [1]. The advent of molecular biology allowed for assessment of molecular signals associated with systemic immune activation. The cytokine tumor necrosis factor (TNF)-alpha was one of the molecular signals associated with anticancer efficacy of innate immune activators such as the Coley vaccine [2]. Studies have demonstrated that TNF-alpha has the ability to induce profound death of cancer cells in vitro and in vivo in animal models, however human studies demonstrated unacceptable levels of toxicity [3-5]. IL-2 was the next cytokine associated with immune activation that was tested. Originally termed T Cell Growth Factor (TCGF) [6], IL-2 was demonstrated in early studies to endow human lymphocytes with ability to selectively kill tumor but not healthy control cells [7]. Subsequent studies have demonstrated that cytotoxic activity was mediated through T cell and natural killer (NK) cells, whose activation requires stimulation of the IL-2 receptor [8,9], which can be accomplished in vivo with high doses of IL-2 [10-12]. Animal studies suggested that IL-2 has a short half-life of approximately 2 minutes after intravenous injection [13,14], and human half life was reported to be approximately an hour [15]. Thus it was apparent that clinical use of IL-2 would be requiring repeated administration at high doses. Despite this pitfall, preclinical studies demonstrated highly potent anti-tumor effect. In 1985 Steven Rosenberg reported regression of established pulmonary metastasis, as well as various subcutaneous tumors by administration of IL-2 [16]. These data were highly promising due to the fact that tumor killing could be achieved systemically, and by activation of specific immune cells that could be identified in vivo as interacting with and inducing death of the tumor.

Early studies of IL-2 demonstrated impressive results in a subset of melanoma and renal cell cancer patients. Development of systemic autoimmunity to melanocytes, such as the occurence of vitiligo during the treatment with systemic IL-2 was found to be predictive of response. These studies were expanded and eventually IL-2 received approval as the first recombinant immunotherapeutic drug by the FDA. There appears to be a dose response with IL-2 in that the doses that seem to be most effective are also associated with significant toxicity. The most significant cause of toxicity is vascular leak syndrome (VSL), manifested as fluid loss into the interstitial space, which is a result of increase vessel permeability. Additional effects include thrombocytopenia, elevated hepatic serum transaminases, hepatocyte necrosis, hypoalbuminemia, tissue and peripheral eosinophilia, and prerenal azotemia [17].

### Vascular leak syndrome

Vascular leak syndrome (VLS) is considered to be the major dose-limiting adverse effect of IL-2 administration. In a meta-analysis of studies performed in metastatic renal cell carcinoma patients, objective responses were observed in 23% of patients, the majority of which lasted more than 10 years [18]. Unfortunately, 65% of patients have to interrupt or stop therapy because of VLS [19,20]. This syndrome is characterized by an increased extracellular fluid extravasation, hypotension, ascites, pulmonary edema, and hydrothorax [21,22], and clinically resembles the systemic inflammatory response syndrome (SIRS). Dermatological manifestations include erythrematous eruptions and mild papillary edema associated with burning and pruritus of the skin [23,24]. Severe forms of VLS are associated with pulmonary or cardiac failure with approximately 1% of treated patients having lethal outcome. Typically the symptoms of VLS are treated by vasopressor therapy and judicious fluid replacement, such as with colloid solutions for their osmotic effects. Patients may also be treated with theophylline and terbutaline, for which clinical experience suggests a possible reduction of the severity and frequency of acute episodes [25].

### Mechanisms of VLS

#### Endothelial activation

At a cellular level it is well-known that VLS is associated with endothelial cell activation and increased with vascular permeability. Biopsies of patients receiving IL-2 revealed an increased expression of adhesion molecules such as ICAM and LFA-1. These proteins are known to promote granulocyte extravasation, however, such upregulation was not observed when IL-2 was added directly to endothelial cell cultures in vitro, suggesting the effect was mediated by other host components [26]. Given that one of the main cellular targets of IL-2 is the T cells, which express two types of IL-2 receptor, it appears that the initial T cell activation is a major contributor to downstream inflammatory effect on endothelium subsequent to IL-2 administration. In an early study, Rosenberg’s group established a murine model for quantifying VLS by administering radioactively iodinated albumin into mice receiving IL-2 and assessing radioactivity of tissues, In this model, increased gamma-counts are correlated with endothelial permeability and leakageof albumin into tissues. They found that administration of IL-2 to nude mice or mice that have been immune suppressed by radiation, cyclophosphamide, or steroids, was associated with markedly reduced or no vascular leakage [27].

#### T cells and nk cells contribute to endothelial activation

It is possible that the process of LAK generation is involved in stimulation of VLS, as was suggested in a study using a similar system in which transfer of LAK along with administration of IL-2 led to more profound endothelial leakage as compared to either alone. Interestingly, in the same study it was shown that depletion of host lymphocytes reduced vascular leakage only in response to IL-2 alone, but not in response to IL-2 and LAK transfer [28]. Other studies have shown that cells bearing the NK marker asialo-GM1 are associated with some of the IL-2 associated toxicities. Anderson et al showed that antiserum to asialo GM1 suppressed mortality, vascular leak syndrome, hepatic damage and reduced infiltration of pulmonary and hepatic vasculature by asialo GM1+ lymphocytes induced by IL-2 treatment. Depletion of the Asialo-GM1 bearing cells did not alter lymphoid hyperplasia, tissue infiltration by Lyt 2+ lymphocytes, tissue and peripheral eosinophilia, or thrombocytopenia. Interestingly, the antisera did not affect the anti-tumor efficacy of IL-2 therapy in BDF mice bearing the colon 38 adenocarcinoma [29]. Thus it is possible that T cell and NK cell activation by the high dose IL-2 induces production of various cytokines, one example being TNF-alpha [30], which are known to induce endothelial cell activation locally [31], and systemically are mediators of SIRS [32].

#### Complement activation

Another event associated with IL-2 administration appears to be complement activation. The complement system is an enzymatic cascade of about 30 circulating proteins, primarily generated by the liver that cause inflammation and amplification of a various immune responses. The complement system can be activated through the classical (antibody mediated) pathway, alternative pathways (antibody-independent), or through the mannose-binding lectin pathway, all of which lead to formation of the membrane attack complex which causes cell lysis through generation of pores in the cell membrane [33]. In a clinical study of metastatic renal cancer patients receiving IL-2 via a 24-hour i.v. infusion at a daily dose of 3 × 10(6) U/m2 for 5 consecutive days, the classical complement pathway components C3 and C4 were measured daily during IL-2 infusion, and after its interruption. IL-2 administration was associated with a significant decrease in both C3 and C4 levels, which normalized on average 5 days after the end of IL-2 infusion [34]. Another study associated presence of VLS in patients receiving IL-2 with complement activation as assessed by levels of C3a and the classical complement component C4a. In this study levels of C3a were as elevated as those found in septic and burn patients [35]. Another study examining 23 cancer patients undergoing therapy with interleukin-2 and lymphokine-activated killer cells demonstrated 3-fold elevations of C3a desArg concentrations by the 8th day of therapy with concentrations of C4a desArg also being elevated by the end of therapy. Associated with activation of the complement system was an increase in the neutrophil cell-surface expression of complement receptor Type 1 and complement receptor Type 3 [36].

This interesting dependence on T cells for complement activation bridges the studies demonstrating that T cells are necessary for both endothelial activation and VLS associated with IL-2 administration. A study by Vachino et al showed that cancer patients had pretreatment similar to control plasma levels of C3a, Ba, Bb, and SC5b-9. Post-IL-2 treatment C3a levels where shown to be increased on average of 15.6-fold (is this differential from controls?). The Ba and Bb proteins, which belong to the alternatively complement activation pathway were augmented 8.0- and 5.0-fold, respectively, subsequent to IL-2 treatment. The plasma levels of the effector complement complex, SC5b-9, was increased 5.0-fold and the plasma C4d and iC3b concentrations increased 4.8- and 2.9-fold, respectively, after treatment. To show the involvement of patient lymphocytes in complement activation, the investigators found that cells expressing the T cell marker CD3 had increased surface expression of anti-C3c and anti-SC5b-9 by 6.2-fold and 5.1-fold, respectively after IL-2 therapy. The authors concluded that the T cells were participating in the IL-2 induced complement activation. This was also demonstrated in that increased concentration of the inflammatory protein C-reactive protein (CRP) was found post-IL-2 therapy [37], and that the T cells bound CRP. T cell bound CRP was capable of activating the alternative complement pathway [38]. Therapeutically, it was demonstrated that administration of the complement inhibitor C1 esterase inhibitor was capable of reducing IL-2 induced hypotension and complement activation in patients [39,40].

#### Complement activates endothelial cells

Various components of the complement cascade have been demonstrated to directly activate endothelial cells, with endothelial cell activation not only causing lymphocyte and neutrophil extravasation, but also thrombosis by the upregulation of tissue factor [41]. C5a is a byproduct of complement activation that has been demonstrated to induce endothelial cell activation and permeability [42-45]. This protein is also a major effector in systemic inflammatory disorders and antibodies to it are being assessed clinically for this condition with some efficacy signals and suppression of endothelial activation published [46,47]. The complement effector complex SC5b-9 was demonstrated in vitro to induce endothelial cell activation via stimulating expression of the Response Gene to Complement (RGC)-32, which in turn activates CDC2 and the AKT pathway [48]. Jeffrey Platt’s group demonstrated that complement activation is associated with induction of IL-1, which in turn stimulates endothelial cells expression of E-selectin, intracellular adhesion molecule-1, vascular cell adhesion molecule-1, Ikappa-Balpha, interleukin (IL)-1alpha, IL-1beta, IL-8, and tissue factor [49]. Thus in the cascade of IL-2 induced VLS, it appears that T cell activation may be associated with complement activation, and complement activation, in turn, stimulates endothelial cell activation. One of the cardinal features of endothelial cell activation is stimulation of the clotting cascade.

#### Coagulation

According to the emerging picture that VLS has many common elements with SIRS, one of the common features is development of local thrombocyte and coagulation system activation. Innate immune response possesses the ability to locally marginalize pathogens by stimulation of clotting and consequent sequestration. However, this process becomes pathological when it occurs at a systemic level, such as SIRS or VLS. Upregulation of tissue factor expression was previously noted on endothelial cells from animals treated with IL-2 [50]. Expression of this protein is known to cause activation of the clotting cascade, as well as stimulate inflammatory processes. Hack et al demonstrated activation of the contact system of coagulation proteins by showing that patients on IL-2 therapy had degradation of factor XII and prekallikrein. Reductions in these proteins appeared not due to protein leakage into the interstitial space, since their levels were still significantly lower, i.e., 80 and 50%, respectively, when corrected for albumin decreases (endothelium may still be more permeable to these clotting factors than to albumin, that’s why i would prefer “appeared”) [51]. Thus it appears that non-specific activation of the coagulation system, and a resulting potential for thrombosis, occur as a result of IL-2 treatment. Given the inherently pro-thrombotic state of many cancer patients, it is theoretically possible that IL-2 therapy may have thrombotic complications, which indeed have previously been reported [52].

#### Granulocytes

Granulocyte activation and tissue infiltration are hallmarks of systemic immune/inflammatory activation. In a study of 4 patients on IL-2, granulocytes became activated following IL-2 treatment with mean peak elastase/alpha 1-antitrypsin (E alpha 1 A) and lactoferrin values of 212 (SEM = 37) and 534 (SEM = 92) ng ml-1 respectively occurring 6 h after the IL-2. Activation of the complement cascade was evidenced by a dose dependent elevation of peak C3a values on day 5 of IL-2. The authors found that there was a significant correlation between C3a levels and the degree of hypotension during the first 24 h after IL-2 (r = 0.91) and parameters of capillary leakage such as weight gain and fall in serum albumin (r = 0.71). The authors concluded that activation of PMN initiates endothelial cell damage which subsequently leads to activation of the complement cascade [53]. Another study showed that neutrophils of patients on IL-2 therapy expressed both phenotypic (up-regulation of CD11b/CD18 adhesion receptor expression) and functional (hydrogen peroxide and hypochlorous acid production) evidence of potent neutrophil activation [54].

#### Gut bacterial translocation

Gut bacterial translocation is associated with chronic inflammatory states such as heart failure [55] and mucositis [56], and acute states such as sepsis [57] or GVHD [58], is translocation of bacterial flora into systemic circulation. Interestingly, IL-2 toxicity is associated with an interference with the gut flora and inflammation. In a recent study, 51 male rats were randomized to receive rIL-2 by intraperitoneal injection at doses (IU) of 10(5) (n = 15), 10(4) (n = 8), 10(3) (n = 8) or 10(2) (n = 8) twice daily, or a saline bolus (n = 12). After 5 days, ileal histomorphology was assessed and the mesenteric lymph node complex was cultured. Results showed that colonisation of mesenteric lymph nodes with Escherichia coli occurred in all rats treated with 10(5) IU of rIL-2, and in 62%, 37% and 12% of rats treated with decreasing doses of rIL-2. No translocation was observed in control animals. An increase in submucosal lymphatics and occasional mucosal disruption was seen only in the group receiving 10(5) IU. These data show that rIL-2 promotes bacterial translocation and suggests a mechanism that may fuel high-dose rIL-2 toxicity in humans [59]. Given the potent effects seen clinically with homeostatically-induced lymphocyte activation [60], and the recent findings that T cell homeostatic proliferation appears to be associated with gut flora translocation [61], it may be possible that tumor suppressive activity of IL-2 may be highly dependent on the gut flora, thus possibly explaining inter-patient variation.

### Oxidative stress

Numerous studies have demonstrated that oxidative stress modifies endothelial cells in a manner that preferentially activates the complement cascade [62]. The involvement of the mannose-binding lectin and the lectin complement pathway (LCP) in promoting complement activation by endothelial cells post oxidative stress was shown in studies using hypoxic (24 hours; 1% O(2))/reoxygenated (3 hours; 21% O(2)) human endothelial cells. Using iC3b deposition as a marker of complement activation, it was shown that N-acetyl-D-glucosamine or D-mannose, but not L-mannose, blocked activation, suggesting that oxidative stress upregulates the mannose dependent pathway. This was also demonstrated using mannose binding lectin deficient serum, as well as antibodies to mannose binding lectin. Furthermore C3 deposition was found in ischemic areas in rats that experienced cardiac ischemia reperfusion injury, a known inducer of oxidative stress [63].

### Vitamin C Depletion after IL-2 therapy

The initial stimulus for writing this review was inspired by the observations made by two of the authors (DTA, CAD) of a scurvy-like condition in a renal cell carcinoma patient treated with IL-2. The patient presented with acute signs and symptoms of scurvy (perifollicular petechiae, erythema, gingivitis and bleeding). Serum ascorbate levels were significantly reduced to almost undetectable levels during the treatment with IL-2 [64]. Although the role of ascorbic acid (AA) hyper-supplementation in stimulation of immunity in healthy subjects is controversial, it is well established that AA deficiency is associated with impaired cell mediated immunity. This has been demonstrated in numerous studies showing that deficiency of this vitamin suppresses T cytotoxic responses, delayed type hypersensitivity, and bacterial clearance [65]. Additionally, it is well-known that NK activity, which mediates IL-2 anti-tumor activity, is suppressed during conditions of AA deficiency [66]. Thus it may be that while IL-2 therapy on the one hand is stimulating T and NK function, the systemic inflammatory syndrome-like effects of this treatment may actually be suppressed by induction of a negative feedback loop. This negative feedback loop with IL-2 therapy was successfully overcome by work using low dose histamine to inhibit IL-2 mediated immune suppression, which led to the “drug” Ceplene (histamine dichloride) receiving approval as an IL-2 adjuvant for treatment of AML [67].

The concept of AA deficiency subsequent to IL-2 therapy was reported previously by another group. Marcus et al evaluated 11 advanced cancer patients suffering from melanoma, renal cell carcinoma and colon cancer being on a 3 phase immunotherapeutic program consisting of: a) 5 days of i.v. high-dose (10(5) units/kg every 8 h) interleukin 2, (b) 6 1/2 days of rest plus leukapheresis; and (c) 4 days of high-dose interleukin 2 plus three infusions of autologous lymphokine-activated killer cells. Mean plasma ascorbic acid levels were normal (0.64 +/- 0.25 mg/dl) before therapy. Mean levels dropped by 80% after the first phase of treatment with high-dose interleukin 2 alone (0.13 +/- 0.08 mg/dl). Subsequently plasma ascorbic acid levels remained severely depleted (0.08 to 0.13 mg/dl) throughout the remainder of the treatment, becoming undetectable (less than 0.05 mg/dl) in eight of 11 patients during this time. Importantly, blood pantothenate and plasma vitamin E remained within normal limits in all 11 patients throughout the phases of therapy, suggesting the hypovitaminosis was specific for AA. Strikingly, responders (n = 3) differed from nonresponders (n = 8) in that plasma ascorbate levels in the former recovered to at least 0.1 mg/dl (frank clinical scurvy) during Phases 2 and 3, whereas levels in the latter fell below this level [68]. Similar results were reported in another study by the same group examining an additional 15 patients [69]. The hypothesis that prognosis was related to AA levels is intriguing because of the possibility of higher immune response in these patients, however this has not been tested.

### AA protects the endothelium from inflammation

The main cause of VSL is increased permeability of the endothelium. Regardless of if the initiating cause is T cell activation, complement, and/or oxidative stress, the effector mechanism of VLS is alteration of endothelial cell function. In SIRS the endothelium is also the main effector causing lethality. Methods by which these cells are altered by both SIRS and IL-2 include: a) endothelial cell apoptosis, b )upregulation of adhesion molecules, and c) increased procoagulant state [70].

It was shown that in vitro administration of AA led to reduction of TNF-alpha induced endothelial cell apoptosis [71]. The effect was mediated in part through suppression of the mitochondria-initiated apoptotic pathway as evidenced by reduced caspase-9 activation and cytochrome c release. Another study examined 34 patients with NYHA class III and IV heart failure who received AA or placebo treatment. AA treatment (2.5 g administered intravenously and 3 days of 4 g per day oral AA) resulted in reduction in circulating apoptotic endothelial cells in the treated but this was not observed in the placebo control group [71]. Various mechanisms for inhibition of endothelial cell apoptosis by AA have been proposed including upregulation of the anti-apoptotic protein bcl-2 [72] and the Rb protein, suppression of p53 [73], and increasing numbers of newly formed endothelial progenitor cells [74].

AA has been demonstrated to reduce endothelial cell expression of the adhesion molecule ICAM-1 in response to TNF-alpha in vitro in human umbilical vein endothelial (HUVEC) cells (HUVEC) [75]. By reducing adhesion molecule expression, AA suppresses systemic neutrophil extravasation during sepsis, especially in the lung [76]. Other endothelial effects of AA include suppression of tissue factor upregulation in response to inflammatory stimuli [77], an effect expected to prevent the hypercoaguable state. Furthermore, ascorbate supplementation has been directly implicated in suppressing endothelial permeability in the face of inflammatory stimuli [78-80], which would hypothetically reduce vascular leakage. Given the importance of NF-kappa B signaling in coordinating endothelial inflammatory changes [81-83], it is important to note that AA at pharmacologically attainable concentrations has been demonstrated to specifically inhibit this transcription factor in endothelial cells [84]. Several pathways of inhibition have been identified including reduction of i-kappa B phosphorylation and subsequent degradation [85], and suppression of activation of the upstream p38 MAPK pathway [86]. In vivo data in support of eventual use in humans has been reported showing that administration of 1 g per day AA in hypercholesterolemic pigs results in suppression of endothelial NF-kappa B activity, as well as increased eNOS, NO, and endothelial function [87]. In another porcine study, renal stenosis was combined with a high cholesterol diet to mimic renovascular disease. AA administered i.v. resulted in suppression of NF-kappa B activation in the endothelium, an effect associated with improved vascular function [88].

### AA Suppresses Systemic Inflammation

The possibility that IL-2 therapy induces a state of systemic inflammation similar to SIRS has been discussed previously. One of the fundamental questions is whether AA actually has beneficial effects on the process of systemic inflammation. A mouse study demonstrated that after challenge with the bacteria Klebsiella pneumonia to induce a sepsis-like state, a 3-fold higher mortality is observed in ascorbate-deficient animals compared to controls [89]. Another study hyper-supplemented animals with AA by administration of 10 mg/kg AA intravenously before induction of sepsis. IV AA treated animals had a 50% survival while only 19% of control animals survived [90]. Other studies demonstrated that hyper-supplementation with AA resulted in better outcomes in sepsis-associated hypoglycemia [91], microcirculatory abnormalities [92], and blunted endothelial responsiveness [93-95] in animal models.

Randomized clinical trials have been performed in septic patients using AA and vitamin E, which demonstrated superior outcomes, as well as reduction in parameters of oxidative stress [96,97]. To date we know of one study in the recent history that assessed AA alone in patients with systemic inflammation. The investigators examined burn patients with > 30% of their total body surface area affected. Patients were administered intravenous AA i.v. (66 mg/kg/hr for 24 hours, n = 19) or received only standard care (controls, n = 18). AA treatment resulted in statistically significant reductions in 24 hr total fluid infusion volume, and fluid retention (indicative of vascular leakage). Perhaps most striking was the decrease in the need for mechanical ventilation: the treated group required an average of average of 12.1 ± 8.8 days, while the control group required 21.3 ± 15.6 days [98]. Given that numerous inflammatory markers associated with VLS are also found in SIRS and severe burn patients, the possibility is presented that AA may exert some beneficial effects on IL-2 therapy, both from the reduction of toxicity perspective, as well as from the stimulation of efficacy.

## Conclusion

We have previously reviewed the rationale for use of IV AA in cancer patients, which includes direct tumor cytotoxicity, antiangiogenic activities, and possibility of increasing quality of life. IL-2 therapy, although one of the oldest successes of cancer immunotherapy, to date, is still not widely implemented due to substantial associated toxicity. To date one drug (Ceplene, histamine dichloride) has succeeded the regulatory and clinical threshold as an agent to reduce IL-2 toxicity and enhance efficacy, thus establishing a regulatory pathway for development of such an agent. In contrast to Ceplene, IV AA is commonly used in the treatment of cancer patients by alternative medicine practitioners, internationally and in the US. Several ongoing FDA clinical trials are currently assessing IV AA as a monotherapy or adjuvant therapy in cancer [99-105]. The rationale that AA preserves endothelial function, inhibits inflammation, and is effective in some states of systemic inflammation, suggests that investigations showed be performed at least in animal models. Our observation of a scurvy-like state induced by IL-2 therapy, the correlation between IL-2 levels and response to immunotherapy, and the fact that IV AA is already clinically used in cancer patients, suggests that translation of the IV AA + IL-2 therapy can be performed in a relatively rapid manner.

## Competing interests

The authors declare that they have no competing interests.

## Authors’ contributions

SCW, BM, NA, BRD, AJK, DTA, CAD, BM, JK, FMM, NHR all contributed to the development of the concept, literature review, discussions, and writing of the manuscript. All authors have read the manuscript and agree to its submission. All authors read and approved the final manuscript.
